# The value of ventricular gradient for predicting pulmonary hypertension and mortality in hemodialysis patients

**DOI:** 10.1038/s41598-021-04186-8

**Published:** 2022-01-10

**Authors:** A. Jaroszyński, T. T. Schlegel, T. Zaborowski, T. Zapolski, W. Załuska, A. Janion-Sadowska, D. Kozieł, S. Głuszek, W. Dąbrowski

**Affiliations:** 1 Collegium Medicum, Jan Kochanowski University in Kielce, Al. IX Wieków Kielc 19A, 25-317, Kielce, Poland; 2 Department of Nephrology, Wojewódzki Szpital Zespolony in Kielce, Kielce, Poland; 3 Department of Molecular Medicine and Surgery, Karolinska Institutet, Stockholm, Sweden; 4 Nicollier-Schlegel Sàrl, Trélex, Switzerland; 5 Department of Family Medicine, Medical University of Lublin, Lublin, Poland; 6 Department of Cardiology, Medical University of Lublin, Lublin, Poland; 7 Department of Nephrology, Medical University of Lublin, Lublin, Poland; 8 Institute of Public Health, Jan Kochanowski University in Kielce, Kielce, Poland; 9 Department of Anesthesiology and Intensive Care, Medical University of Lublin, Lublin, Poland

**Keywords:** Cardiology, Nephrology

## Abstract

Pulmonary hypertension (PHT) is associated with increased mortality in hemodialysis (HD) patients. The ventricular gradient optimized for right ventricular pressure overload (VG-RVPO) is sensitive to early changes in right ventricular overload. The study aimed to assess the ability of the VG-RVPO to detect PHT and predict all-cause and cardiac mortality in HD patients. 265 selected HD patients were enrolled. Clinical, biochemical, electrocardiographic, and echocardiographic parameters were evaluated. Patients were divided into normal and abnormal VG-RVPO groups, and were followed-up for 3 years. Abnormal VG-RVPO patients were more likely to be at high or intermediate risk for PHT, were older, had longer HD vintage, higher prevalence of myocardial infarction, higher parathormone levels, shorter pulmonary flow acceleration time, lower left ventricular ejection fraction, higher values of left atrial volume index, left ventricular mass index, and peak tricuspid regurgitant velocity. Both all-cause and CV mortality were higher in abnormal VG-RVPO group. In multivariate Cox analysis, VG-RVPO remained an independent and strong predictor of all-cause and CV mortality. In HD patients, abnormal VG-RVPO not only predicts PHT, but also all-cause and CV mortality.

## Introduction

Pulmonary hypertension (PHT) is prevalent in hemodialysis (HD) patients, and is associated with increased morbidity and mortality. The high incidence of PHT in HD patients relates not only to left-sided heart disease, but also to HD-specific factors. The deterioration of kidney function per se may be a trigger for the development of PHT, and the uremic milieu leads to pulmonary vasoconstriction and decreased compliance of the pulmonary vasculature^1–7^. Mechanisms responsible for PHT in HD patients have not been completely understood. However, abnormal endothelium-dependent vasodilatation, vascular calcification, thromboembolic disease, hypervolemia, increased pulmonary vascular flow due to the presence of arterio-venous fistulas, anemia and sleep-disordered breathing play a role^7–10^. The classical symptoms of PHT occur relatively late and may be confounded by overhydration in HD patients, leading to the delayed diagnosis and the worsening of prognosis^11^. To enable earlier detection of PHT, regular screening by use of noninvasive tools is advisable. However, the diagnostic accuracy of standard electrocardiographic (ECG) parameters is low in detecting PHT^10,12–16^.

Recently, there has been renewed interest in the ventricular gradient (VG) to quantify ventricular action potential duration (APD) heterogeneity. The spatial VG is the vectorial sum of spatial QRS and spatial T integral vectors. VG runs from the area of the longest mean APD toward the area of myocardium having the shortest APD. Owing to this fact VG is considered a potential marker of increased CV risk, including sudden death. Changes in magnitude and/or orientation of the VG reflect APD inhomogeneities related to electrical RV remodelling as a result of an increased RV pressure load. Previously published studies have demonstrated that spatial VG, and especially ventricular gradient optimized for right ventricular pressure overload (VG-RVPO) was a useful CV predictor in some clinical settings^16–27^. VG-RVPO is the VG vector sum in a specific spatial direction providing an optimized projection for detection of RV pressure overload (VG magnitude at elevation 27° and azimuth 155°)^16,23,25–27^. Several studies have revealed that VG-RVPO can accurately detect increased pulmonary pressures, is sensitive to early changes in RV overload, and has higher diagnostic accuracy for elevated RV pressure than other known ECG-derived parameters^14,16,19,20,25,27,28^. However, the prognostic value of VG-RVPO has not been evaluated in HD patients.

The purpose of the study was to assess the ability of VG-RVPO to detect PHT and predict all-cause as well as cardiac mortality in HD patients.

## Material and methods

### Participant recruitment and selection

This prospective study was conducted in adult HD patients treated at two HD units in Lublin (Poland). Exclusion criteria applied in the present study were as follows: treatment < 3 months (to include exclusively ESRD patients); advanced neoplastic disease; acute coronary syndrome or stroke over the prior 3 months (to exclude the influence of acute ischemia on VG-RVPO values); electronic pacing; or symptoms of acute infection or hemorrhage at baseline (to reduce the possible influence of transient factors on VG-RVPO values).

Owing to the fact that it was impossible to estimate population size meeting the criteria described above, a prospective sample size calculation was not performed. All HD patients in Lublin were invited and those who decided to participate were included. Written informed consent was obtained from all participants. The study was approved by the Ethical Committee of Medical University of Lublin (KE-0254/125/2011) and conducted in accordance with the Declaration of Helsinki.

### Control group

ECG was performed in 50 gender- (26 F and 24 M) and age-matched (69.3 ± 6.1 years) healthy volunteers who had normal laboratory tests and displayed no abnormalities detected by physical examination and chest x-ray.

### Biochemical parameters

Routinely determined biochemical parameters were measured at the beginning of the follow-up (the day after the HD session) at the same time as both echocardiography and electrocardiography. The following parameters were evaluated: sodium, potassium, calcium, phosphorus, creatinine, urea, hemoglobin, parathormone (PTH), total protein, albumin, C-reactive protein (CRP), total cholesterol, HDL cholesterol, LDL cholesterol, triglycerides (TG) and troponin T. Blood was obtained in the morning after at least 8 h fasting.

### Electrocardiographic parameters

**Figure 1:**
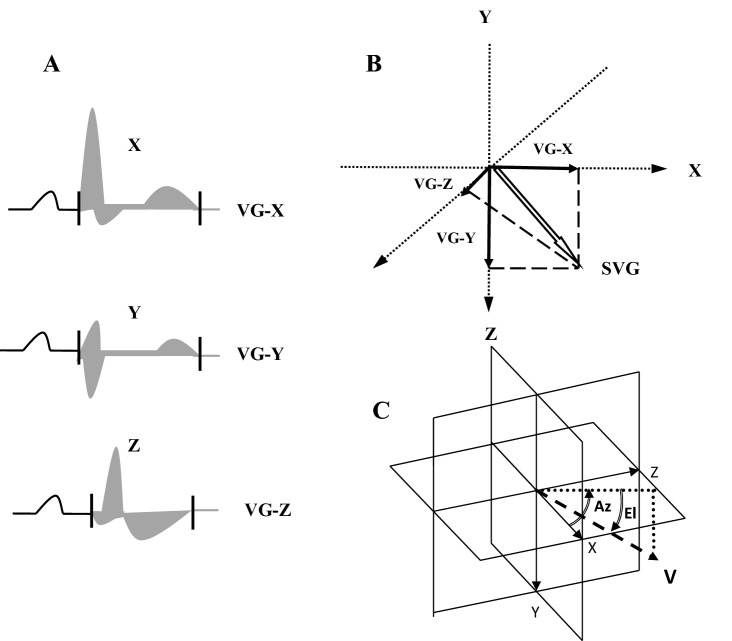
(**A**) The QRS- and T-area vectors are calculated from the areas under the curves in the QRST x, y, and z complexes. (**B**) The ventricular gradient (VG) is the resultant vector of the QRS and T integral vector. (**C**) VG-RVPO the VG vector sum in a specific spatial direction providing an optimized projection for detection of RV pressure overload (VG magnitude at elevation 27° and azimuth 155°). Az, azimuth; El, elevation; V, heart vector; T, transverse palne; F, frontal plane; S, sagital plane. Measurements were performed using MEDEA device (licence number 245/2018, http://medea.pl/komputerowy-aparat-ekg/).

Surface 12-lead resting ECG was recorded in each patient and in controls using a MEDEA device (Kardio PCM-u, Poland). ECGs were recorded in an electrically shielded and noise-proof room with subjects lying in the horizontal position after at least 5 min rest. All recordings were obtained during the short, inter-dialysis interval. The 10 s recordings were automatically averaged to a single beat, and transformed into three orthogonal leads X, Y and Z using the inverse Dower method. The areas under the curves of the QRST complexes in X-, Y-, and Z leads were calculated automatically by integrating voltages over the entire QRST complex from the MEDEA software (licence number 245/2018, http://medea.pl/komputerowy-aparat-ekg/). VG-RVPO was calculated using trigonometric equation: VG-RVPO = -0.807526151172 * VGx + 0.404508497187 * VGy + 0.376555628452 * VGz where VGx, VGy and VGz are the areas under the X-, Y-, and Z-components of the VCG, respectively (Fig. 1).

### Echocardiographic examination

Transthoracic echocardiography was performed in the morning after dialysis sessions^29,30^ using a 2.5–3.5 MHz probe and Sonos 5500 and 7500 units (Philips, Andover, MA, USA) according to American Society of Echocardiography recommendations^30–32^. The experienced cardiologist who performed echocardiography was blinded to all other clinical data. According to the guidelines of echocardiographic assessment of PHT, peak tricuspid valve velocity (TRV) was measured as the key parameter in determining the probability of PHT^13,32,33^. TRV was measured by continuous wave Doppler. The optimal window at parasternal short axis (PSAX), A4C view, subcostal view or a modified view between the PSAX and A4C was obtained. Then measurement from that complete tricuspid regurgitation envelope that had the highest velocity was performed. An average of three beats in sinus rhythm and five beats in atrial fibrillation was calculated. A TRV < 2.8 m/s was considered normal. In addition to TRV measurements, echocardiographic parameters suggestive of PHT originating from the heart ventricles, pulmonary artery, and inferior vena cava and right atrium were assessed. These included, for example, the pulmonary flow acceleration time (ACT). Patients were then divided into low, intermediate, and high risk PHT groups based on their TRV values and the presence or absence of echocardiographic parameters mentioned above suggesting PHT^13,32,33^.

In addition to the echocardiographic parameters necessary to assess the probability of PHT, other echocardiographic parameters known to be prognostic factors in HD patients, such as left ventricular mass index (LVMI), left ventricular ejection fraction (LVEF), left atrial volume index (LAVI), and ratio between early mitral inflow velocity and mitral annular early diastolic velocity (E/e`), were also measured^30,31^.

### Follow-up data

Patients were followed for 36 months or until the date of death or renal transplant starting from the day of baseline assessment. The end points of the current study were defined as all-cause and CV mortality. The definition of CV death was in line with that presented in Standardized Definitions for End Point Events in Cardiovascular Trials ^34^. Events were determined by two independent physicians blinded to other results. If opinions were divergent, the event was verified by the third physician, an expert in cardiology.

### Statistical analysis

Statistical analysis was performed by using Statistica Version 10 as described in detail previously^35^. Initially the data were assessed for distribution by using Kolmogorov–Smirnov test. When normally distributed, continuous variables were expressed as mean ± SD, and as median and range when non-normally distributed. Categorical data were expressed as frequencies and percentages. For analysis, patients were divided into two groups depending on the VG-RVPO values. The VG-RVPO value ≥ − 13 mV*ms was considered abnormal and < − 13 mV*ms was considered normal. The cut-off value was based on previous research results^23,24,27^. Student’s t-test for independent variables was used in the case of normally distributed parameters. The Mann -Whitney U-test was used when results from at least one of the groups were not normally distributed. Linear regression analysis was performed using the Pearson or Spearman test, as appropriate. Multiple stepwise regression analysis was performed to include within the model only those measures that significantly differed (*p* value < 0.05) between normal and abnormal VG-RVPO groups. The Kaplan–Meier method was used to assess the value of VG-RVPO for predicting all-cause and cardiac mortality in HD patients. Differences between patient groups were assessed using the log-rank test. A Cox proportional hazard regression analysis was also performed wherein only those variables with *p* value < 0.15 in the univariate analysis were considered for inclusion in the multivariate Cox model. Statistical significance was defined as *p* < 0.05.

## Results

### Patients’ clinical characteristics

Of the 303 available HD patients, 38 were excluded due to: treatment < 3 months (n = 8), advanced neoplastic disease (n = 7), acute coronary syndrome or stroke over the period of 3 months (n = 4), pacing (n = 11), and acute infections or hemorrhages (n = 8).

The remaining 265 HD patients (143 females and 122 males), aged 32–87 years (mean 70.2 ± 8.04) were included. The mean HD vintage was 45.93 ± 21.11 (from 3 to 156) months. The causes of renal failure were as follows: diabetes (n = 103), chronic glomerulonephritis (n = 73), hypertensive nephropathy (n = 22), polycystic kidney disease (n = 10), obstructive nephropathy (n = 11), chronic pyelonephritis (n = 7), and unknown/unsure (n = 39). Clinical baseline characteristics of the studied patients are depicted in Table 1.

**Table 1:** Baseline characteristics of patients.

Parameter	All patients n = 265	VG-RVPO ≥ − 13 mV*ms n = 81	VG-RVPO < − 13 mV*ms n = 184	*p*
Age (years)	70.2 ± 8.34	72.4 ± 8.18	69.3 ± 8.21	** < 0.001**
Sex (F/M – n)	1.18	1.17	1.18	0.763
HD vintage (months)	45.9 ± 21.11	57.1 ± 18.65	41.8 ± 19.31	** < 0.001**
MI (%)	21.9	28.4	19.0	**0.001**
Diabetes mellitus (%)	51.9	50.6	54.3	0.234
Hypertension (%)	58.9	58.0	59.2	0.653
Smoking	10.2	8.6	10.8	0.196
Beta-blockers (%)	83.4	86.4	82.1	0.227
ACEI/ARB (%)	71.3	74.0	70.1	0.314
Statins (%)	53.9	55.5	53.3	0.299
E/e`(n)	14.51 ± 5.11	15.19 ± 4.99	13.96 ± 5.11	0.195
LVMI (g/m^2^)	145.2 ± 41.12	156.1 ± 36.09	141.4 ± 38.76	** < 0.001**
LVEF (%)	55.13 ± 5.11	51.56 ± 5.11	56.64 ± 5.22	** < 0.001**
TRV (m/s)	2.49 ± 0.67	3.69 ± 0.70	1.92 ± 0.65	** < 0.001**
ACT (ms)	101.6 ± 27	74.5 ± 24	113 ± 25	** < 0.001**
LAVI (ml/m^2^)	36.75 ± 8.04	39.03 ± 7.64	35.86 ± 7.72	**0.001**
High PHT risk (%)	20.0	54.3	4.9	< 0.001
Intermediate PHT risk (%)	35.5	45.7	31.0	< 0.001
Low PHT risk (%)	44.5	0.0	64.1	< 0.001
Hemoglobin (g/dL)	11.03 ± 1.08	10.91 ± 1.01	11.10 ± 1.22	0.569
Total cholesterol (mg/dL)	187.1 ± 36.71	188.1 ± 36.22	186.9 ± 35.14	0.681
LDL cholesterol (mg/dL)	117.3 ± 29.65	116.1 ± 29.15	118.3 ± 30.01	0.524
HDL cholesterol (mg/dL)	44.02 ± 17.35	45.1 ± 16.18	44.00 ± 16.55	0.723
Triglycerides (mg/dL)	173.4 ± 59.13	171.0 ± 56.86	174.2 ± 56.5	0.435
PTH, range (pg/mL)	415 (0.0–1278)	487 (0.0–1278)	402 (0.0–1006)	** < 0.001**
Albumin (g/dL)	3.69 ± 0.31	3.55 ± 0.32	3.73 ± 0.30	0.116
CRP, range (mg/dL)	8.15 (0.31–95.1)	9.74 (0.31–61.2)	7.59 (0.91–95.1)	0.207
Troponin T, range (μg/L)	0.043 (0.00–0.702)	0.061 (0.035–0.702)	0.040 (0.00–0.597)	0.132
Sodium (mmol/L)	137.5 ± 2.63	137.9 ± 2.56	137.4 ± 2.79	0.871
Potassium (mmol/L)	5.43 ± 0.72	5.58 ± 0.74	5.37 ± 0.63	0.342
Calcium (mmol/L)	2.46 ± 0.22	2.48 ± 0.24	2.45 ± 0.25	0.389
Phosphate (mmol/L)	2.27 ± 0.27	2.30 ± 0.25	2.25 ± 0.24	0.307

MI, myocardial infarction; ACEI, inhibitors–angiotensin converting enzyme inhibitors; ARB, angiotensin 2 receptor blockers; E/e`, ratio between early mitral inflow velocity and mitral annular early diastolic velocity; LVMI, left ventricular mass index; LVEF, left ventricle ejection fraction; TRV, peak tricuspid regurgitant velocity; AcT, pulmonary flow acceleration time; LAVI, left atrial volume index; PHT, pulmonary hypertension; PTH, parathormone; CRP, C-reactive protein.

Out of 265 patients enrolled in the study, 71.3% were treated with angiotensin-converting enzyme inhibitors (ACEI) or angiotensin receptor blockers (ARB), 83.4% received beta-blockers, and 53.9% statins. Hypertension was present in 58.9% of patients, history of myocardial infarction in 21.9%, and left ventricular hypertrophy (LVH) in 65.3%.

### VG-RVPO comparison between patients and controls

VG-RVPO was less negative in patients compared with controls (− 20.72 ± 16.35 mV∙ms vs. − 31.15 ± 8.62 mV∙ms, *p* < 0.001). No difference was found between VG-RVPO in HD females and males (− 20.98 ± 14.33 vs.—20.57 ± 15.13, *p* = 0.737).

### Differences between patient groups

Patients in the abnormal VG-RVPO group were more likely to be at high or intermediate risk for PHT (*p* < 0.001 in both cases), and none of the patients from the abnormal VG-RVPO group was at low risk for PHT (*p* < 0.001). Only 4.9% of the patients from the normal VG-RVPO group were at high risk for PHT. Patients in the abnormal VG-RVPO group were older (*p* < 0.001), had longer HD vintage (*p* < 0.001), and higher prevalence of MI (*p* = 0.001). With regard to biochemical indices, the abnormal VG-RVPO group also had higher PTH levels (*p* < 0.001). In relation to echocardiographic parameters, patients in the abnormal VG-RVPO group had shorter AcT (pulmonary flow acceleration time) (*p* < 0.001), lower LVEF values (*p* < 0.001), higher LAVI (*p* = 0.001), higher LVMI (*p* < 0.001), and higher TRV values (*p* < 0.001).

On the basis of echocardiographic criteria patients were divided into 3 groups according to PHT risk (low, intermediate and high). Differences in VG-RVPO values were found between the high and low risk groups (− 6.9 ± 13.9 vs. − 25.9 ± 16.4, *p* < 0.001), and the high and intermediate risk groups (− 6.9 ± 13.9 vs. − 18.2 ± 15.1, *p* < 0.001). The difference between the intermediate and low risks groups was also significant, however less pronounced (− 18.2 ± 15.1 vs. − 25.9 ± 16.4, *p* = 0.018). Likewise, differences in AcT values were found between the low and high risk groups (109.1 ± 21 vs. 82.02 ± 22.73, *p* < 0.001), and between the low and intermediate groups (109.1 ± 21 vs. 96.3 ± 20.1, *p* = 0.017). The difference in ACT between intermediate and high risk groups did not reach significance (96.3 ± 20.1 vs. 109.1 ± 21, *p* = 0.119). Moreover, VG-RVPO correlated negatively with TRV (r =—0.682), *p* < 0.001) and positively with ACT (r = 0.611, *p* < 0.001).

### Survival and multivariate analysis

During the mean follow-up period of 26.9 ± 4.8 months, 99 all-cause deaths were noted (37.5%). The mortality rate was 12.5% per year. Twenty three patients were transplanted. In the abnormal VG-RVPO group the incidence of all-cause death was 61.7% and was higher than that of the normal VG-RVPO group (26.6%, *p* < 0.001).

CV death contributed to 49.5% of all deaths. The proportion of CV death in the abnormal VG-RVPO group (72.1%) was higher than that in the normal VG-RVPO group (31.3%, *p* < 0.001). Out of 21 transplanted patients only 2 came from the abnormal VG-RVPO group.

**Figure 2:**
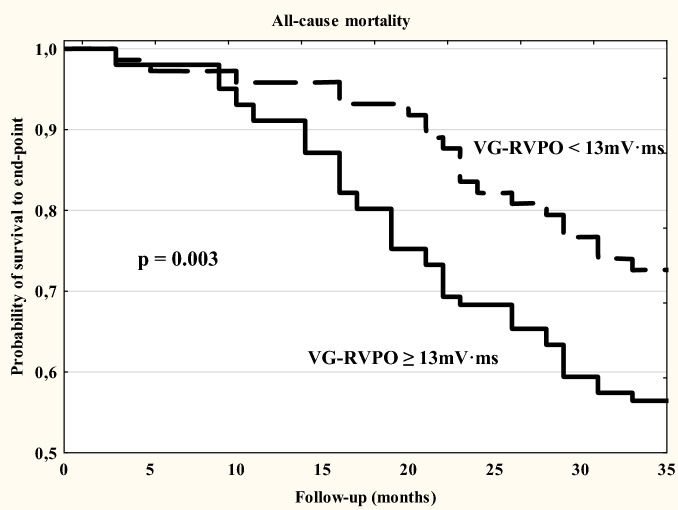
Survival plots analysis for all-cause mortality in HD patients stratified by normal and abnormal VG-RVPO value.

**Figure 3:**
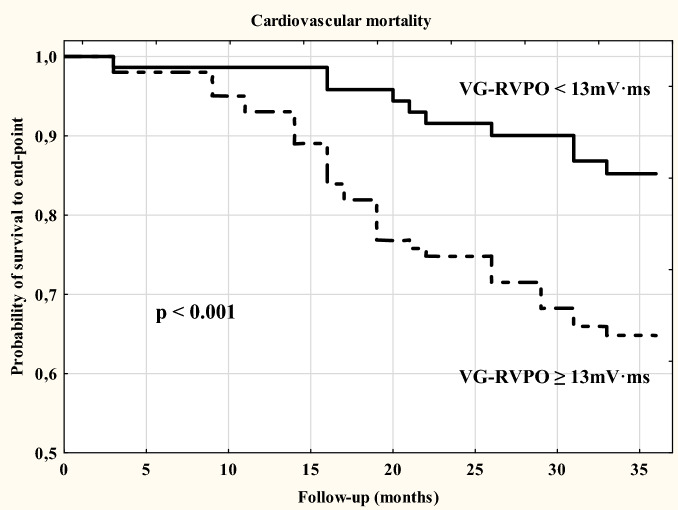
Survival plots analysis for cardiovascular mortality in HD patients stratified by normal and abnormal VG-RVPO value.

By Kaplan–Meier analysis, the cumulative incidence of both all-cause and CV mortality was higher in the abnormal VG-RVPO group then the normal VG-RVPO group; (-log-rank, *p* = 0.003 and *p* < 0.001, respectively (Figs. 2 and 3).

Multivariate Cox proportional hazard regression analyses were performed using a model consisting of univariate predictors of cardiac mortality to control for possible confounders. The results of both univariate and multivariate Cox proportional hazard regression analyses are presented in Tables 2 and 3. A multivariate analysis revealed that age [hazard ratio (HR) 1.76, *p* < 0.001], TRV (HR 1.37, *p* = 0.018], LVMI (HR 1.79, *p* = 004), and VG-RVPO (HR 2.01, *p* = 0.002) were independent predictors of all-cause mortality. In the case of CV mortality, multivariate Cox proportional analysis indicated age (HR 2.19, *p* < 0.001), HD vintage (HR 1.76, *p* = 0.006), TRV (HR 1.71, *p* = 0.019), LVMI (HR 1.72, *p* = 0.007), LAVI (HR 1.77, *p* = 0.011) and VG-RVPO (HR 2.40, *p* < 0.001) as independent predictors of CV mortality.

**Table 2:** Uni- and multivariate predictors of all-cause mortality.

Parameter	Univariate HR (95% CI)	*P*	Multivariate HR (95% CI)	*p*
Age	1.85 (1.36–2.51)	*P* < 0.001	1.76 (1.19–2.63)	< 0.001
HD vintage	1.49 (0.84–1.99)	0.008	1.21 (0.58–2.13)	0.212
History of MI	1.69 (0.85–2.51)	0.063	1.37 (0.64–2.273)	0.102
TRV	1.42 (0.91–2.11)	0.002	1.37 (0.71–2.54)	0.018
AcT	0.85 (0.28–1.88)	0.031	0.91 (0.41–2.49)	0.167
LVMI	2.26 (1.77–2.65)	< 0.001	1.79 (1.11–2.79)	0.004
EF	0.82 (0.49–1.67)	0.021	0.90 (0.52–1.73)	0.118
LAVI	1.87 (1.17–2.65)	0.016	1.37 (0.95–3.82)	0.266
PTH	2.31 (1.18–3.97)	0.174		
VG-RVPO	2.29 (1.83–2.73)	< 0.001	2.01 (1.64–2.70)	0.002

HR, hazard ratio; CI, confidence interval; HD, hemodialysis; MI, myocardial infarction; TRV, peak tricuspid regurgitant velocity; AcT, pulmonary flow acceleration time; LVMI, left ventricular mass index; LVEF––left ventricle ejection fraction; LAVI—left atrial volume index; PTH, parathormone; VGx, ventricular gradient projected on the x-axis. In the multivariate analyses, parameters with a *p* ≤ 0.15 were entered.

**Table 3:** Uni- and multivariate predictors of cardiovascular mortality.

Parameter	Univariate HR (95% CI)	*p*	Multivariate HR (95% CI)	*p*
Age	2.33 (1.52–2.92)	< 0.001	2.19 (1.58–2.97)	< 0.001
HD vintage	1.68 (0.94–2.15)	0.001	1.76 (0.74–2.56)	0.006
History of MI	1.54 (0.71–2.26)	0.008	1.35 (0.59–2.47)	0.101
TRV	1.91 (1.04–2.66)	0.004	1.71 (0.99–2.96)	0.019
AcT	0.81 (0.33–1.94)	0.146	0.93 (0.31–2.14)	0.215
LVMI	1.95 (1.17–2.30)	< 0.001	1.71 (0.94–2.62)	0.007
EF	0.86 (0.41–1.82)	0.002	0.84 (0.31–2.46)	0.099
LAVI	2.14 (1.15–3.64)	0.018	1.77 (1.19–2.94)	0.011
PTH	2.71 (1.45–3.76)	0.012	2.14 (1.26–3.58)	0.044
VG-RVPO	2.70 (2.11–3.17)	< 0.001	2.40 (1.71–2.99)	< 0.001

HR, hazard ratio; CI, confidence interval; HD, hemodialysis; MI, myocardial infarction; TRV, peak tricuspid regurgitant velocity; AcT, pulmonary flow acceleration time; LVMI, left ventricular mass index; LVEF—left ventricle ejection fraction; LAVI—left atrial volume index; PTH, parathormone. In the multivariate analyses, parameters with a *p* ≤ 0.15 were entered.

## Discussion

The two major findings of the present study were that: (1) VG-RVPO corresponds with PHT and correlates with echocardiographic parameters related to PHT; and (2) VG-RVPO is an independent predictor of both all-cause and CV mortality in HD patients.

In the present study VG-RVPO values were higher in HD patients compared to controls, and 55.5% of patients were classified into intermediate and high risk groups for PHT based on echocardiographic parameters. This suggests that PHT is prevalent in HD patients, a finding in agreement with previous studies demonstrating that the prevalence of PHT is around 18.8–68.8%, depending on the criteria adopted for PHT diagnosis, selection factors and co-morbidities^2,5,8,10,36,37^. Right heart catheterization was not included in the study protocol because of medical-ethical reasons. We were therefore unable to estimate the prevalence of PHT but instead only the risk of PHT. Given that echocardiography is only a screening tool for PHT^32,33^, our results should therefore be further corroborated in studies using RV catheterization.

To our knowledge this is the first study to demonstrate the usefulness of VG-RVPO in PHT prediction in HD patients. In the present study VG-RVPOcorresponded with PHT and correlated with echocardiographic parameters related to PHT, suggesting its usefulness in PHT screening and/or monitoring. Given that clinical symptoms of PHT are nonspecific and often masked by the presence of overhydration, pulmonary and heart diseases, the diagnosis of PHT is often delayed in HD patients, worsening prognosis^11^. While RV catheterization is the “gold standard” of PHT diagnosis, simple, inexpensive, noninvasive diagnostic tests are needed to allow earlier detection of PHT. However, conventional surface ECG lacks diagnostic accuracy. PHT is characterized by increased RV pressure overload, and hence, increased RV wall tension which only over time can cause RV hypertrophy^38^. Thus anatomical remodeling of the heart is not needed for development of PHT, and PHT can also occur without RV hypertrophy. Myocardial action potentials change when RV pressure changes, due to mechanoelectrical feedback. This results in VG-RVPO changes reflecting RV pressure load. Given that standard ECG criteria rely on depolarization characteristics of hypertrophic RV, that may not yet have developed, unless and until RV hypertrophy occurs, the value of standard ECG parameters in early detection of increased pulmonary pressure is limited^12,15,16,20,28^.

The usefulness of VG-RVPO in assessing PHT in HD patients as determined in our study is in line with recently published papers, which have demonstrated that abnormal VG and especially VG-RVPO is strongly associated with PHT both in experimental^20,28^ and clinical studies^16,23–28,38,40^. The clinical usefulness of VG-RVPO in PHT detection was confirmed in scleroderma^23,24^, acute pulmonary embolism^27^, and patients with suspected PHT^25^. Additionally, VG-RVPO correlated strongly with pulmonary artery pressure^25^, and was suitable as a monitoring tool reflecting pulmonary artery pressure changes in patients with PHT^38^. Moreover, in a group of patients with scleroderma VG-RVPO was superior to echocardiography derived parameters for predicting increased RV load as well as to detect disease progression in early phases of the disease^24,26^.

Our study suggests that VG-RVPO is an independent and strong predictor of both all-cause and CV mortality in HD patients. This finding is in line with previous observations that the VG is a risk predictor both in the general population^38^ and in post-infarction patients^22^. The spatial VG has also been associated with sudden cardiac death in the general population^19^. Abnormal VG-RVPO was also associated with increased mortality in patients with scleroderma, and the spatial VG vector projected on the x-axis predicted all-cause mortality in PHT patients^14^.

The association between VG-RVPO and all-cause as well as CV mortality is probably due to the fact that VG-RVPO, like some other VG-based parameters, is a measure of APD heterogeneity, which is associated with increased susceptibility to ventricular arrhythmias. It is considered to be a marker of global electrical heterogeneity of the myocardium^19,20,39^. Any change in the myocardial electrical heterogeneity is associated with a change in VG-RVPO value. RV pressure load affects ventricular APD distribution resulting in a change in VG-RVPO^16,19,20,40^. Though VG-RVPO is the risk stratifier, especially suitable in PHT prediction, the knowledge about its physiologic correlates is limited and requires further study.

If our results are confirmed in future studies using right heart catheterization, VG-RVPO may become a useful tool for both the prediction and serial monitoring of PHT in HD patients.

## Limitation

Our study has some limitations. First, we have evaluated exclusively echocardiographic parameters predictive of PHT but did not perform right heart catheterization, the gold standard in the diagnosis of PHT. We are aware of the limitations of echocardiography as a screening tool for PHT. However, echocardiography is accepted as the most useful screening tool for PHT^32,33^, and we recognize that without RV catheterizations, our results should only be construed as preliminary. Moreover, the fact that echocardiography was performed by only one cardiologist in our study may be a source of potential bias. Second, it is likely that serial rather than single VG-RVPO measurements may influence the results, making VG-RVPO either more or less useful in predicting PHT as well as clinical events in HD patients. Third, the inverse Dower transform utilized by our MEDEA device is not necessarily the scientifically most optimal transform for derivation of the X, Y and Z leads. Therefore, a more optimized transform and also a more fully automated software method would ideally be used in future studies to further optimize results and eliminate potential methodological bias^41^.

## Conclusions

In HD patients, abnormal VG-RVPO not only predicts PHT, but also all-cause and CV mortality.
